# Emergency Laparotomy Risk Assessment: A Qualitative Study of General Surgeons and Trainees

**DOI:** 10.1111/ajr.70075

**Published:** 2025-07-25

**Authors:** Joseph N. Hewitt, Thomas J. Milton, Christopher Dobbins, Markus I. Trochsler

**Affiliations:** ^1^ The University of Adelaide, Discipline of Surgery, the Queen Elizabeth Hospital Woodville South Australia Australia; ^2^ Department of Surgery Royal Adelaide Hospital Adelaide South Australia Australia

**Keywords:** emergency laparotomy, general surgery, risk assessment

## Abstract

**Objective:**

General surgeons perform emergency laparotomies on a heterogeneous patient population. Scoring tools have been developed to quantify the risk of mortality after EL, but the uptake of these tools is poor. We aimed to characterise the attitudes of surgeons to risk assessment tools.

**Setting:**

Australia.

**Participants:**

General surgeons, registrars and residents.

**Design:**

Semi‐structured interviews with participants, analysed using Framework Method.

**Results:**

Fifteen participants were interviewed. Barriers identified included perceived lack of utility, competing priorities, unit or hospital culture, individual surgeon attitudes, lack of funding, junior medical staff turnover, and lack of familiarity. Potential strategies for improvement identified included education, integration with electronic health records, and prompting at time of theatre booking.

**Conclusion:**

Our findings will be of interest to those undertaking quality improvement work with risk assessment. This is important given recommendations for universal risk assessment but the low uptake of risk assessment in practice.

**Summary**

- What is already known on this subject
  ○ Emergency laparotomy preoperative risk assessment is recommended by peak professional bodies and provides benefits to patient consent, clinician decision making, and system logistics.
  ○ Despite recommendations, risk assessment is applied far from universally.
  ○ Survey based data has shown clinicians choose which risk assessment tool to use based on ease of use.
- What this paper adds
  ○ This is the first study to report interview‐based data regarding risk assessment advantages and barriers to use.
  ○ This paper adds novel knowledge of clinicians thinking around preoperative risk assessment and barriers to implementation.
  ○ The findings have implications for quality improvement projects aimed at improving risk assessment rates.

## 1 Introduction

Emergency laparotomy (EL) is a procedure commonly performed by general surgeons. Patients undergoing EL range from infants to the elderly and present with a wide range of pathology and comorbidity. EL also carries a high risk of mortality. Estimates range from 7.1% in‐hospital mortality in the Australian context [1] to 9.2% 30‐day mortality in UK estimates [2]. For complex elective surgery on multi‐comorbid patients, the rural practitioner has the option to refer patients to quaternary centres. In contrast, the emergency nature of EL often necessitates complex and high‐stakes abdominal surgery being undertaken in a rural centre which otherwise might usually only provide simple surgical services. Previous research has demonstrated comparable outcomes between patients undergoing EL in rural and metropolitan centres [3].

Given the high mortality associated with EL and the heterogeneity in outcomes, various large‐scale quality improvement projects have been instigated to improve these outcomes. These include the UK's National Emergency Laparotomy Audit (NELA) and the Australia and New Zealand Emergency Laparotomy Audit (ANZELA). Both NELA and ANZELA benchmark against various standards for EL including whether patients have a pre‐operative assessment of risk of mortality performed and documented [1, 2]. In the UK, auditing adherence to these standards has resulted in a reduction in 30‐day mortality from 13% to 9% over 8 years of the project [2]. NELA standard adherence is linked to hospital funding via a “Best Practice Tariff.” The target for a hospital to receive additional funding is 85% of patients undergoing preoperative risk assessment [4]. Adherence to standards is far from universal in the Australian context. The most recent ANZELA report states a pre‐operative risk assessment rate of 52% [5] for participating hospitals whereas our study of South Australian metropolitan and regional hospitals showed only 10% of patients undergoing EL had a risk assessment documented [6]. Adherence to risk assessment recommendations is particularly important in view of previous research suggesting decreased mortality rates in those undergoing pre‐operative risk assessment [7]. Apart from this, risk assessment can aid with informed consent discussions with patients. Mortality ought to be considered a material risk of any EL and hence discussion of the risk of mortality would be a legal expectation of the consent process. Apart from this risk assessment may also determine appropriate post‐operative destination and help decide urgency of an operation or even whether to proceed at all. Risk assessment in the rural hospital can also aid in decisions of whether a patient should be transferred to a quaternary centre preoperatively or have upfront surgery.

Despite these benefits, the listing of risk assessment as a standard by NELA and ANZELA, and the widespread availability of multiple risk assessment tools for emergency laparotomy [8], usage remains far from universal in Australia. Previous survey‐based research in New Zealand has shown that clinicians choose which risk assessment tool to use based on ease of use and familiarity, and that a greater emphasis on quality of life is desired by clinicians rather than on mortality [9]. There is a paucity of further data on barriers to the use of risk assessment tools. Accordingly, we carried out a qualitative semi‐structured interview‐based study investigating current EL risk assessment practices, barriers to using risk assessment tools, and potential strategies for improvement.

## 2 Methodology and Methods

We undertook semi‐structured interviews with general surgeons, general surgery trainees and junior doctors working in general surgery in metropolitan and regional hospitals. The methodology and results are reported in accordance with the consolidated criteria for reporting qualitative research (COREQ) [10]. The study received approval from an appropriate human research ethics committee. All participants provided informed consent prior to participation.

### 2.1 Sampling and Participant Recruitment

We used a snowball purposive sampling approach to recruit participants for interviews. We identified potential participants through prior knowledge of those with the greatest involvement in emergency general surgery on‐call rosters at our hospitals and those with strong opinions on risk assessment. Participants were then asked to identify other potential participants after undergoing an interview. Participants were approached through a combination of face‐toto‐face, telephone, and email. A sample size of 15 was chosen based on known thresholds for data saturation in qualitative studies [11] and pragmatically.

### 2.2 Interview Setting, Questions and Focal Points

Interviews were undertaken by a mixture of face‐to‐face, phone call and video conferencing with Microsoft Teams. Interviews lasted approximately 20 min and were audio recorded for later transcription. An overall template of questions and focal points is provided in Appendix A, noting that the template evolved during the research process based on identification of new themes from concurrent analysis of previously collected data. Interviews were conducted by the first author, who is male and at the time was working as a surgical registrar. The first author had previous training in qualitative research methods through master's degree study. Some participants had a pre‐existing working relationship with the first author. Only the first author and participants were present during interviews.

### 2.3 Analysis and Reporting

The first author transcribed the data using Descript (San Francisco, CA). The first author coded the data following interviews using the Framework Method [12]. The second author independently coded two transcripts to confirm the coding framework in discussion with the first author. Questions regarding themes derived from the data following each interview were incorporated into future interviews if relevant.

## 3 Results

Fifteen participants were interviewed, consisting of six consultants, four fellows, three registrars, and two residents. Eleven participants were male. Themes, the views expressed by participants, and representative quotes are shown in Table 1.

**TABLE 1:** Themes, participants' views and exemplifying quotes.

Theme	Views expressed	Quotes
Current practice	Participants current practice ranged from universal use of risk assessment tools to no use at all Of those that did use risk assessment tools, NELA was commonly mentioned	“I've been using the NELA score every time. That's probably the one that I find most accessible and easy to use. Yesterday morning it took me 2 min to get a score.” (consultant) “I never did a NELA score from myself thinking it should be done. I was never proactive with it. The times that NELA scores were completed was because the consultant or fellow asked me to do it.” (resident) “I don't use the scores very much at all.” (consultant) “The honest answer is no I don't use them. Sometimes the registrars tell me about, you know, the different score is this but to be honest I've never asked about one or made one myself.” (consultant)
Perceived benefits of risk assessment	Benefits cited included aiding informed consent, deciding seniority of operator, urgency of operation, post‐operative destination, benefits to research or audit	“I've found it can often make a confusing situation a little bit more transparent to the patient and give them solid numbers.” (registrar) “If they are high risk, you know, you really have the evidence which suggests that you need to have a consultant involved in the actual operation and not delegate that operation and just be remote.” (consultant) “It also helps me determine when it's suitable for a registrar to be doing the case.” (consultant) “But I have used it where I've had to have discussions with ICU to say, look, no, this patient does need to come to you. Or with surgeons elsewhere, no, we should not be transferring this patient. Or with anaesthetics saying, look, we need to do this case now not later.” (consultant) “I think in the research and quality improvement context it's very useful because then you know what your burden is compared to the hospital you're comparing with so it provides a way of propensity matching risks.” (consultant)
Perceived barriers to risk assessment	Barriers cited included lack of utility, time pressures or competing priorities, lack of standardisation, unit culture, individual surgeon preference, junior staff turnover, lack of familiarity or ability with risk assessment tools	“I don't think it makes or breaks the decision. I think it's more like… ‘I don't think they should have surgery, and by the way, the NELA score is really bad!’” (consultant) “If it was really very valuable for decision making, then I don't think that we would have a problem. Like you won't find a patient gets to the operating theatre without getting bloods done, right?” (consultant) “One of the biggest problems is the reason it's not done is the registrars have better things to be doing.” (consultant) “Probably because it's not standard routine practice so there's no understanding as to who should do it and when should it be done. And because it's not routine practice, it gets forgotten. And then when it's remembered, it's, you know, it depends on who remembers it. It's not like, have you given antibiotics? It's not like your time out [surgical safety checklist].” (consultant) “At least for me it kind of does come back to the culture of the unit that you're working on and how involved the consultant is on promoting preoperative risk assessment. Well actually probably more consultant specific. Some bosses don't really care but [particular surgeon] always asks about it.” (registrar) “That's one of the challenges of getting the risk assessment score done routinely is that we do have an itinerant workforce, essentially, with junior staff coming in and out.” (consultant) “So for a junior person to estimate things that NELA ask for like blood loss is very challenging. Or peritoneal soiling. Or whether malignancy is anticipated. Or the urgency of the operation maybe we don't quite get the subtleties.” (resident)
Strategies for improvement	Strategies suggested included integration with electronic medical records or automation of risk assessment calculation, standardisation, prompting or mandates, education	“It should be like eGFR you know? You've got a creatinine and there it is. All the data is on the computer already. I think at least the EMR [electronic medical record] could help prompt it more, or when you book a theatre case there could be more prompting there.” (registrar) “We need to have a system wide agreement that this is part of a standard practice protocol. So anybody going for emergency laparotomy must have a NELA score. And then the second thing that must happen is there needs to be visibility. So, it needs to be a prompt. Just like you have the surgical safety checklist.” (consultant) “I think theatre staff could have a role in prompting it. So, even if you're a newbie coming on to a rotation, that'll be a prompt or somebody asking you, ‘What about this?’” (fellow) “I think we can get better with something education based. The more people know about it and see some value in it, the more likely it will be that more senior clinicians will ask about it. I'm sure it doesn't always need to be the stick for the juniors as well, if you can sort of turn it as a carrot, you know, because it helps you to present your patient well that might be the other option.” (consultant)

### 3.1 Current Risk Assessment Practice

Risk assessment practices of participants varied across the entire spectrum of universal use to never having used risk assessment tools previously. NELA, POSSUM, and ACS‐NSQIP tools were mentioned specifically by participants. Surgeons spoke about using risk assessment calculators quickly and easily on their phones, usually at the time of seeing the patient, while discussing with a colleague or when booking a patient for theatre. Consultants expressed frustration with junior staff turnover, which hampers increased usage as they expect risk assessment to be completed by the admitting registrar or resident, and junior doctor rotations mean there is a constant need for re‐orientation and education in this area. Registrars and residents, in contrast, feel that risk assessment is largely driven by consultant staff or unit culture, and in some cases would even decide to omit or carry out a risk assessment based on prior knowledge of the surgeon on call's preferences. Consultants and registrars commented that they used risk assessment tools in informed consent discussions with patients and their families, and some commented they document this on the operative consent form in line with NELA standards.

### 3.2 Perceived Benefits of Risk Assessment

Participants cited various benefits of risk assessment prior to laparotomy. Aiding in informed consent discussions was a benefit for many, noting a concrete number to encapsulate risk was something patients appreciated or found useful in decision making. Both consultants and fellows commented that a risk assessment was useful in deciding whether they should personally perform an operation or whether a registrar could be allowed to. Interestingly, no registrars or residents commented on a risk assessment changing their view as to involving senior staff, presumably because consultants are more universally involved in the decision to take a patient to the operating theatre. Participants spoke about how a risk assessment could aid discussions with other specialists or registrars, help in decisions of the appropriate post‐operative destination for the patient, and facilitate benchmarking between patients or hospitals in the audit or research process. Participants highlighted the benefit of risk assessment in particular for patients who might undergo EL in a regional hospital. Especially where resources are stretched or EL is out of the usual day‐to‐day practice for a hospital or a surgical team, risk assessment was felt to be doubly helpful in guiding practitioners through the decision‐making process around EL.

### 3.3 Perceived Barriers to Risk Assessment

There was a view that risk assessment tools lack utility from participants, with some seeing a recommendation for their use as actively wasting the time of busy clinicians with multiple competing priorities. Some saw risk assessment as something to pay lip service to simply because it is listed as a recommendation by NELA and ANZELA but not something that is useful in its own right. Some again saw risk assessment as only useful when it would clearly confirm their already firmly held opinion and would only undertake a risk assessment when planning to recommend against operation or in favour of palliation, to give them more “ammunition” in patient or family discussion. Time pressures and competing priorities were a recurring theme, and risk assessment falls by the wayside when an admitting registrar has already accumulated several more referrals in the time it took to see and formulate a plan for the patient undergoing EL. As previously mentioned, consultants stressed the impact of junior medical staff turnover on rates of risk assessment, with new batches of junior staff every three or six months then needing reminding or training in using risk assessment tools. In contrast, junior clinicians stressed the impact of the culture of a unit and the preferences of individual consultants on whether risk assessment was completed or not. Junior clinicians also spoke about difficulties completing risk assessments when they felt they did not have the requisite clinical judgement to accurately appraise a situation.

### 3.4 Strategies for Improvement

Several strategies to improve rates of pre‐operative risk assessment were suggested by participants. Integration with electronic medical records or at least partial automation of risk assessment calculation represents low hanging fruit for improvement. With a large amount of the work of admitting a patient including documentation, prescribing and theatre booking occurring with the use of electronic medical records, risk assessment could be streamlined into this process. The “number of clicks” to calculate a risk could be decreased when physiological and biochemical data which are available within the electronic medical record already, are integrated automatically. Standardisation of protocols and setting expectations was mentioned as another avenue for improvement, as was increased prompting, possibly at the time of theatre booking or mandates, although clinicians did voice some concern about added burdens with this. Finally, increased education especially for junior staff was thought to be another factor which would improve rates of risk assessment.

## 4 Discussion

The heterogeneity in risk assessment practices among participants reflects the nuanced challenges associated with implementing standardised protocols in emergency laparotomy. While some clinicians demonstrated consistent utilisation of risk assessment tools such as NELA, POSSUM, and ACS‐NSQIP, others displayed a lack of proactive engagement or even opposition to their use. Many risk assessment tools are available and the number available possibly contributes to the lack of universal use given the lack of a known gold standard tool. For example, the SORT tool is one recommended by the Australian and New Zealand College of Anaesthetists (ANZCA) although this was not mentioned by any of our participants. Possibly, there is a difference in preference for risk assessment tools between the surgical population we studied and other professional craft groups. Strikingly, there is also a disconnect between consultant and junior staff attitudes towards where responsibility lies in improving rates of risk assessment. This discrepancy highlights the need for targeted interventions to promote a culture of accountability and ensure the routine integration of risk assessment into clinical practice.

The observed frustration among consultants regarding junior staff turnover underscores the importance of continuity and consistency in training and education initiatives. High turnover rates necessitate ongoing re‐orientation and training, posing challenges to the sustained implementation of risk assessment protocols. Instituting standardised training programs and providing robust support systems for junior clinicians can help mitigate these challenges and foster a more cohesive approach to risk assessment across healthcare settings. In regional settings especially, surgical teams are likely to be small and hence handover and maintenance of ‘institutional knowledge’ is doubly important. This observed frustration also however potentially represents a lack of consultant leadership in this area. As one participant in our study observed, no EL patient will make it to the operating theatre without having bloods taken. If consultants placed similar value and emphasis on risk assessment as on blood results, then this would doubtless go some way towards improving risk assessment rates.

The perceived benefits of risk assessment in facilitating informed consent discussions and guiding clinical decision‐making underscore its potential to enhance patient‐centred care and improve surgical outcomes. By providing patients and their families with transparent risk estimates, clinicians empower them to make informed decisions regarding treatment options and expected outcomes. Furthermore, risk assessment enables clinicians to stratify patients based on risk, guiding decisions regarding the level of senior involvement and post‐operative management. In regional or rural settings especially, risk assessment will also guide decisions of preoperative transfer or upfront surgery and maintain previous comparability of outcomes between metropolitan and rural centres.

However, despite these benefits, several barriers to the routine use of risk assessment tools were identified. Time constraints emerged as a significant impediment, with clinicians often prioritising competing demands over risk assessment. This underscores the need for streamlined processes and the integration of risk assessment tools into electronic medical records to optimise efficiency and minimise workflow disruptions. As noted by one participant, however, calculating a risk assessment may take less than 2 min with the aid of a smartphone and thus time pressure may not be the largest barrier to risk assessment. Additionally, addressing misconceptions regarding the clinical utility of risk assessment and providing clinicians with adequate training and support can help overcome resistance and promote broader acceptance of risk assessment practices.

The proposed strategies for improvement, including automation of risk assessment calculations, standardisation of protocols, and enhanced educational efforts, represent critical steps towards optimising risk assessment processes in emergency laparotomy. By leveraging technology and implementing evidence‐based practices, healthcare organisations can streamline workflows, enhance accuracy, and improve patient outcomes. Participants in our study did not identify one potential strategy for improvement of risk assessment rates which has seen particular success in the UK, being incentivisation of risk assessment rates through linkage to hospital funding.

Maybe surprisingly, our study did not identify dissatisfaction with risk assessment tools use of mortality as the primary outcome of interest as a reason they were not used. This is in contrast to a previous NZ‐based survey of surgeons, anaesthetists, and intensivists which showed a preference for including quality of life outcomes rather than mortality as the outcome of interest [9]. There are likely issues with quantifying quality of life; however, in what is already a complex and underused process, further complications may decrease uptake.

Our study has some limitations. The small sample size may limit generalisability. Likewise, the population studied being drawn from Australia will limit generalisability also. Further survey‐based research might provide additional insights. Further research could extend beyond surgeons and trainees to others involved in the patient's journey through an emergency laparotomy, such as anaesthetists, intensivists, nursing staff, and patients themselves. This could provide different perspectives on the benefits of risk assessment, barriers to its use, and potential strategies for improvement. It is possible that surgeons are not the best judges of the actual barriers to risk assessment and strategies to improve that, as we have investigated in this study. Indeed, our findings represent the opinions of studied participants and do not provide evidence regarding the validity of each of these points. We have already criticised participant complaints of the time taken to perform risk assessment and of consultants regarding junior staff turnover or not taking responsibility for risk assessment.

Our research will be of interest to those undertaking an audit of risk assessment rates for EL and those seeking to improve those rates. We have discussed various attitudes towards risk assessment as well as perceived benefits and barriers, and suggested strategies to improve the uptake of risk assessment for EL. Further research should be directed at evaluating the success of suggested strategies in improving risk assessment rates and monitoring outcomes alongside that.

## Author Contributions

The author takes full responsibility for this article.

## Ethics Statement

The study received approval from the Central Adelaide Local Health Network Human Research Ethics Committee (reference 16 081).

## Conflicts of Interest

The authors declare no conflicts of interest.

## Data Availability

The data that support the findings of this study are available on request from the corresponding author. The data are not publicly available due to privacy or ethical restrictions.

## Appendix A

### Semi Structured Interview Question Template

Can you tell me how emergency laparotomy features in your practice?

How do you decide how risky an operation will be?

Looking at a patient in emergency or on the ward, how can you tell there might be challenges during and after the operation?

Do you use any risk assessment tools? How often?

Are you familiar with the tools available?

Do risk assessment tools offer any advantage over a surgeon's clinical judgement?

What would the ideal risk assessment tool take into account?

What problems are there with risk assessment tools?

Would anything convince you to use one?

Is there a reason you choose to use one particular tool or the other?

What factors do the risk assessment tools not take into account?

What are the most important factors you take into account?

Does anything frustrate you about using risk assessment tools? What are they missing?

Would anything convince you to use them more often?

Can you tell me about a time a laparotomy went badly unexpectedly? Did you use a risk calculator?

Is there anything you would like to add on the topic?
